# Functional determinants of gate-DNA selection and cleavage by bacterial type II topoisomerases

**DOI:** 10.1093/nar/gkt696

**Published:** 2013-08-10

**Authors:** Elisa Arnoldi, Xiao-Su Pan, L Mark Fisher

**Affiliations:** Division of Biomedical Sciences, St.George’s, University of London, London SW17 0RE, UK

## Abstract

Antibacterial fluoroquinolones trap a cleavage complex of gyrase and topoisomerase (topo) IV inducing site-specific DNA breakage within a bent DNA gate engaged in DNA transport. Despite its importance for drug action and in revealing potential sites of topoisomerase catalysis, the mechanism of DNA selectivity is poorly understood. To explore its functional basis, we generated mutant versions of the strongly cleaved E-site and used a novel competitive assay to examine their gemifloxacin-mediated DNA breakage by *Streptococcus pneumoniae* topo IV and gyrase. Parallel studies of Ca^2+^-induced cleavage distinguished ‘intrinsic recognition’ of DNA cleavage sites by topo IV from drug-induced preferences. Analysis revealed strong enzyme-determined requirements for −4G, −2A and −1T bases preceding the breakage site (between −1 and +1) and enzyme-unique or degenerate determinants at −3, plus drug-specific preferences at +2/+3 and for +1 purines associated with drug intercalation. Similar cleavage rules were seen additionally at the novel V-site identified here in ColE1-derived plasmids. In concert with DNA binding data, our results provide functional evidence for DNA, enzyme and drug contributions to DNA cleavage at the gate, suggest a mechanism for DNA discrimination involving enzyme-induced DNA bending/helix distortion and cleavage complex stabilization and advance understanding of fluoroquinolones as important cleavage-enhancing therapeutics.

## INTRODUCTION

Type II DNA topoisomerases are biologically essential enzymes that mediate the ATP-dependent gating of one DNA duplex through a transient double-stranded break introduced in another DNA duplex (1–3). They function to regulate chromosomal DNA supercoiling and to remove supercoils and catenanes produced variously in DNA replication, transcription and recombination. Eukaryotes express the homodimeric topoisomerase II found as alpha and beta isoforms in mammalian cells (4). Topoisomerase (topo) IV and DNA gyrase are the bacterial type II topoisomerases each performing specialized cellular roles (5–8). Topo IV promotes the unlinking of catenated daughter chromosomes to allow their segregation at cell division, whereas gyrase introduces negative supercoils into DNA, facilitating replication fork movement and allowing control of gene expression (9). For each enzyme, the active complex is a tetramer composed of two copies both of topo IV ParC and ParE subunits or of gyrase GyrA and GyrB proteins (2). The subunits have a modular design with the closely related N-terminal regions of the ParC and GyrA proteins constituting the DNA breakage–reunion domains and the divergent C-terminal ends directing inter- and intra-molecular DNA transport, respectively (2,8,10,11). The highly conserved ParE and GyrB subunits have N- and C-terminal domains comprising, respectively, the ATPase site that directs conformational changes in the complex and the TOPRIM fold that binds Mg^2+^ ions essential for reversible DNA breakage (2,10–15).

In common with other type II topoisomerases, topo IV and gyrase transiently break DNA through the formation of a covalent enzyme–DNA intermediate known as the ‘cleavage complex’ (11,16). The DNA in this complex (sometimes termed the gate-DNA or G-segment) exhibits a 4-bp staggered break involving the covalent linkage of ParC (GyrA) subunits, one to each 5′-phosphate end *via* active-site tyrosine residues. The cleavage complex allows DNA transport through the gate-DNA coordinated with the sequential opening and closure of other enzyme gates formed by the N-terminal ends of ParE (GyrB) and within the ParC (GyrA) subunits. DNA breakage is readily reversed by nucleophilic attack of the 3′OH ends on the phosphotyrosyl links regenerating the intact DNA backbone. Indeed, the DNA breakage–reunion equilibrium of type II topoisomerases lies well over to the sealed state avoiding the inadvertant release of lethal double-stranded DNA breaks (11,16).

**Figure 1.**
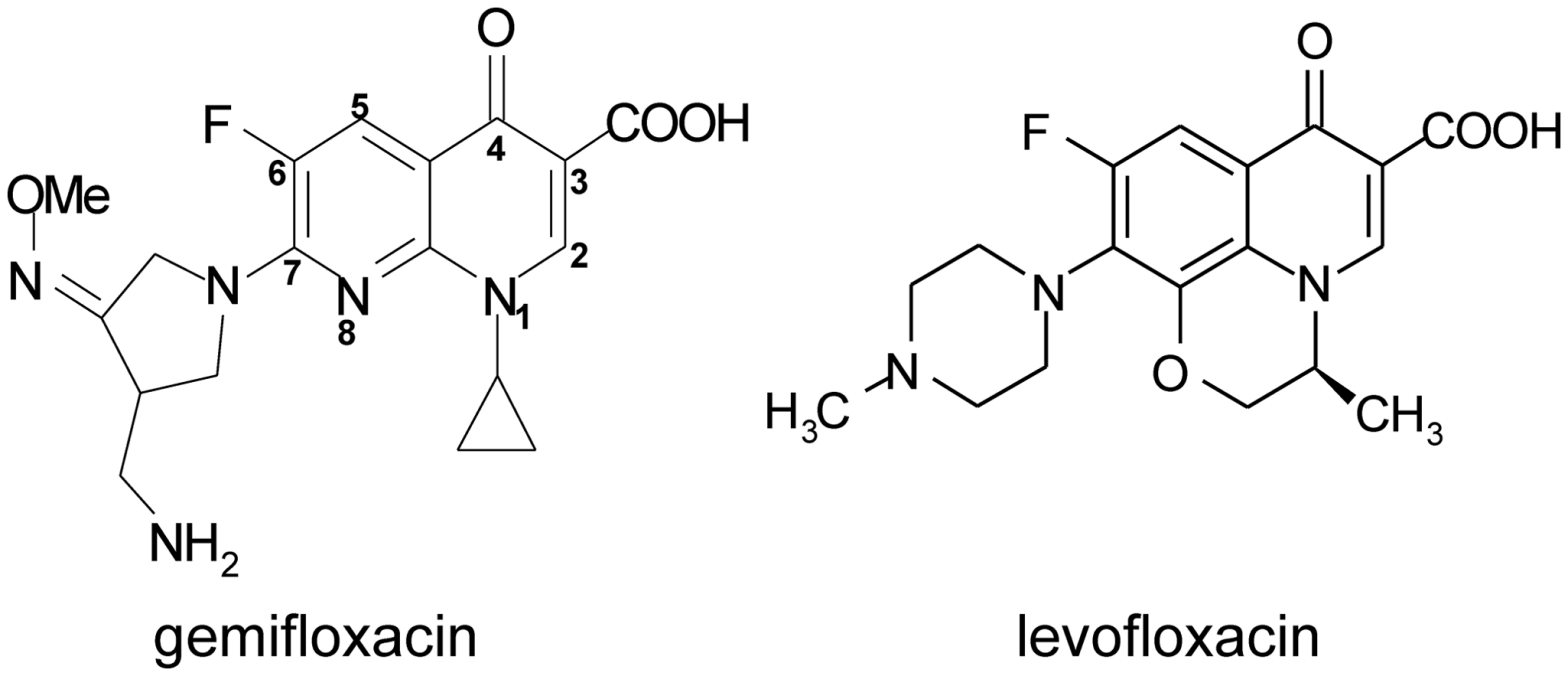
Structures of gemifloxacin and levofloxacin, two antipneumococcal fluoroquinolones.

A variety of clinically important antibacterial and anticancer drugs inhibit type II topoisomerases, sustaining interest in these enzymes as therapeutic targets (17,18). Anticancer agents such as etoposide target eukaryotic topo II, whereas antibacterial fluoroquinolones, e.g. gemifloxacin and levofloxacin, target topo IV and/or gyrase (Figure 1). The drugs interfere with DNA religation to stabilize a topoisomerase cleavage complex, which cellular processes convert into a cytotoxic lesion, possibly a double-stranded DNA break (19). Drug-arrested complexes are readily detected *in vivo* and *in vitro* by denaturation with sodium dodecyl sulphate (SDS), releasing double-stranded DNA breaks at specific sites (20,21). Although anticancer drug-promoted DNA cleavage has been extensively studied for eukaryotic topo II (22), much less is known about fluoroquinolone-mediated breakage by bacterial type II topoisomerases.

Consensus cleavage sequences have been derived for topo IV and gyrase from *Streptococcus pneumoniae* in the presence of gemifloxacin and other clinically important quinolones used against this Gram-positive pathogen (Figure 1) (23). Using stringent statistical criteria, these sequences were G(G/c)(A/t)a*GNNCt(T/a)N(C/a) and GN_4_G(G/c)(A/c)G*GNNCtTN(C/a) for topo IV and gyrase, respectively (where the asterisk indicates the cleavage site between −1 and +1 bases, capital letter denotes preferred base, lower case letter denotes disfavoured base and N, no base preference). A second analysis using SV40 template DNA instead of pneumococcal DNA and weaker statistical stringency gave a different outcome, notably a preference for GGGCCC at positions −1 to +5 possibly reflecting the greater representation of GGG and CCC traits in SV40 DNA (24). Template bias, enzyme and drug levels, inclusion of weak sites, averaging across potentially different binding modes and the stringency adopted can all affect the derived consensus. Moreover, consensus data leave open a number of unanswered questions. First, what constitutes an ‘optimal’ cleavage site? Second, do gyrase and topo IV have the same or different cleavage requirements? Third, are all consensus elements necessary for efficient cleavage? Fourth, what distinguishes ‘cleavable’ and ‘uncleavable’ sequences? Finally, how are sequences discriminated and what molecular interactions are involved?

To address these questions, we have used the complementary approach of manipulating strongly cleaved sites by systematic mutagenesis. Cleavage-enhancing, -disrupting and -neutral changes define the contacts needed for efficient DNA breakage. Bacteriophage T4 topoisomerase provides the best example to date, but this specialized enzyme more closely resembles a eukaryotic type II topoisomerase, for example, in requiring anticancer drugs for efficient DNA cleavage (25). Few studies have focused on prokaryotic type II topoisomerases *per se*. An early investigation of DNA cleavage selectivity by *Escherichia coli* gyrase was limited in scope by the restricted mutagenesis techniques and poor potency of the quinolones then in use (26). Furthermore, previous studies have lacked high-resolution X-ray structure information needed for mechanistic analysis.

Here we establish the functional determinants for quinolone-induced DNA breakage by pneumococcal topo IV and gyrase at the strongly cleaved E- and (newly discovered) V-sites, the first systematic analysis for any bacterial type II enzyme. To facilitate analysis, we developed a powerful new assay that allows rapid screening of multiple sites in competition. By overlaying the sequence preferences we observed in Ca^2+^-induced DNA breakage by topo IV, we could distinguish enzyme versus drug-specific determinants. Guided by recent E-site-topo IV structures revealing two quinolones intercalated at a highly bent cleaved DNA gate (27,28), we propose a model that accounts for DNA selectivity in cleavage.

## MATERIALS AND METHODS

### Bacterial strains, reagents and plasmids

*Escherichia coli* XL1-Blue supercompetent cells and ‘One Shot TOP10’ chemically competent *E. coli* were obtained from Stratagene and Invitrogen. *Escherichia coli* strain BL21 (λDE3) pLysS was obtained from Novagen. Oligonucleotide primers were synthesized by Sigma and Metabion. Gemifloxacin and levofloxacin were from GlaxoSmithKline, Harlow, UK and from McNeil-Ortho, respectively. Other quinolones were from our laboratory stocks. The 4–12% polyacrylamide Tris-Borate-EDTA (TBE) gels were from Invitrogen. Ni-NTA resin was from Qiagen. SYBR® Green I Nucleic Acid Gel Stain was obtained from Lonza, Slough, UK. BIOTAQ™ DNA polymerase was from Bioline and Pfu DNA polymerase was from Stratagene. Plasmid pXP1, carrying a 4.3-kb HindIII fragment of the *S. pneumoniae parE-parC* locus (29) was from our laboratory strain collection. Plasmid vector pCR2.1 TOPO was supplied by Invitrogen. Supercoiled pBR322 was from New England BioLabs. [γ-^33^P]ATP (3000 Ci/mmol) was purchased from MP Biomedicals NV/SA in 9.25 MBq amounts.

### Recombinant topo IV and gyrase subunits

*Streptococcus pneumoniae* ParC, ParE, GyrA and GyrB subunits were expressed as His-tagged proteins in *E. coli* BL21 (λDE3) pLysS and purified to >95% homogeneity as previously described (30,31).

### Mutagenesis of the topo IV E- and V-sites

The E-site from the *S. pneumoniae parE* gene contained in a 256-bp polymerase chain reaction (PCR) product was amplified from plasmid pXP1 (29) by PCR using BIOTAQ™ DNA polymerase with forward primer E-FOR, 5′ GATTCTCAGATAACATTCTA and reverse primer E-REV, 5′ TAGGGGGCTTCCTAGTTTA. The PCR reaction (50 µl) contained 67 mM Tris–HCl (pH 8.8 at 25°C), 16 mM (NH_4_)_2_SO_4_, 0.01% stabilizer, 1.5 µl of 50 mM MgCl_2_, 0.5 µl of 100 mM dNTP mix, 125 ng each of forward and reverse primers, 5 ng of DNA template and BIOTAQ™ polymerase (5 units). Conditions for amplification were as follows: denaturation at 95°C for 30 s, annealing at 53°C for 35 s and extension at 74°C for 1 min, 30 cycles. The PCR product was purified on a QIAquick spin column (Qiagen) according to the manufacturer’s instructions, ligated into the pCR2.1 TOPO vector and used to transform competent *E. coli* TOP10 cells. Recombinant clones were selected by plating on agar plates containing ampicillin. Individual colonies were picked and 5 ml of overnight cultures were used to purify plasmid DNA using the QIAprep Miniprep method according to the manufacturer’s instructions. DNA sequence analysis confirmed the cloning of the correct sequence and one plasmid clone, pEA1, was used for subsequent studies.

Plasmid pEA1 was used as a template to generate a variety of mutated versions of the E-site using the QuikChange site-directed mutagenesis kit (Stratagene) and complementary 42-mers viz 5′TACCAAGGTCATGAATGACTATGCACGTAAAACAGGTCTTC (top strand) and 5′GAAGACCTGTTTTACGTGCATAGTCATTCATGACCTTGGTA (bottom strand) bearing appropriately placed mutations in the E-site sequence underlined. Mutated pEA1 plasmids were recovered and the presence of the correct mutations was verified by DNA sequence analysis of the 256-bp E-site insert.

Mutations at the V-site were introduced similarly using plasmid pEA1 (EM4C) (bearing −4C/+8G changes at the E-site) as template and two pairs of mutagenic primers:

VM4C-F (5′CACTCAAAGGCGGTA**C**TACGGTTATC**G**ACAGAATCAGGGGAT) and VM4C-R (5′ATCCCCTGATTCTGT**C**GATAACCGTA**G**TACCGCCTTTGAGTG) specifying −4C/+8G changes (bold underlined), and VM2T-F (5′CACTCAAAGGCGGTAAT**T**CGGTTA**A**CCACAGAATCAGGGGAT) and VM2T-R (5′ATCCCCTGATTCTGTGG**T**TAACCG**A**ATTACCGCCTTTGAGTG) carrying −2T/+6A changes. The mutant plasmids were recovered and V-site mutations were confirmed by DNA sequence analysis.

### DNA cleavage assays

A variety of DNA substrates was used in cleavage reactions with recombinant pneumococcal topo IV and gyrase including PCR products and linear plasmid DNA. In initial studies, wild-type (wt) and mutant E-site substrates were amplified by PCR as 256-bp fragments using pEA1 or its mutant derivatives as template with BIOTAQ™ polymerase and E-FOR and E-REV primers under the conditions described above. DNA products were recovered using QIAquick PCR purification columns and the DNA concentration and purity was determined spectrophotometrically. Each PCR product (0.2 µg) was incubated with *S. pneumoniae* ParC/GyrA (0.45 µg) and ParE/GyrB (0.85 µg) in the presence or absence of quinolones. Topo IV reaction buffer contained 40 mM Tris–HCl, pH 7.5, 6 mM MgCl_2_, 10 mM DTT, 200 mM potassium glutamate, and 50 µg/ml bovine serum albumin (BSA) in total volume of 20 µl. Gyrase buffer comprised 35 mM Tris–HCl, pH 7.5, 6 mM MgCl_2_, 1.8 mM spermidine, 24 mM KCl, 5 mM DTT, 36 µg/ml BSA, and 6.5% glycerol (w/v). After incubation at 37°C for 1 h, SDS and proteinase K were added to final concentrations of 1% and 100 µg/ml. Incubation was continued for another hour at 42°C to digest ParC/GyrA protein covalently bound to DNA. Samples were run in 4–12% gradient polyacrylamide gels in TBE buffer, visualized by staining with ethidium bromide and photographed under UV illumination using a Biomolecular Imager (Fujifilm).

For competitive cleavage assays, wt and mutant E-site PCR products were amplified as 276-bp and as 256-, 236- and 216-bp products using forward primer E-FOR and the reverse primers ER-20 A (5′AAAAAAAAAAAAAAAAAAAATAGGGGGCTTCCTAGTTTA), EREV (5′GATTCTCAGATAACATTCTA), ER2 (5′-CCTTGGTCTGTCCTTCAAAC) and ER4 (5′-TGCAAGTGTTCTTCAGGAAC), respectively. PCR products were purified on spin columns and quantified as described previously. Cleavage reactions with topo IV were carried out as above with gemifloxacin or levofloxacin in the absence or presence of 1 mM ATP using a mixture of 100 ng each of the wt E-site and three different mutant E-sites present on 276-, 256-, 236- and 216-bp PCR fragments, respectively. After addition of SDS and incubation with proteinase K, substrates and cleavage products were separated on 4–12% polyacrylamide TBE gels as above. After staining with ethidium bromide or SYBR Green, DNA bands were photographed and quantitated using Fuji Multi Gauge software.

Plasmid pEA1 and its derivatives bearing mutated E- and V-sites were linearized with ScaI and used as substrates for DNA cleavage by topo IV. The cleavage assay contained linear plasmid DNA (450 ng), ParC (450 ng), ParE (850 ng), 6 mM MgCl_2_ and gemifloxacin at 2.5 µM contained in the same topo IV cleavage buffer used for cleavage of E-site PCR fragments. Reactions were incubated at 37°C for l h, and following SDS and proteinase K treatment, DNA products were separated by electrophoresis in a 1.2% agarose gel run in TBE. DNA bands were visualized by staining with ethidium bromide and photographed under UV light.

For calcium cleavage experiments, quinolone and Mg^2+^ were omitted and substituted with 32 mM CaCl_2_.

### DNA sequence analysis of V- and E-site cleavage

Gemifloxacin-mediated cleavage of Nco-I linearized pEA1 revealed a second major cleavage site, the V-site, that produced 1.9- and 2.2-kb cleavage fragments. Use of other restriction fragments located the site just outside the replication origin region in the vector sequence of pCR2.1 TOPO. The V-site was found to reside on a 457-bp fragment amplified from pEA1 plasmid by PCR using the BIOTAQ™ polymerase enzyme and oligonucleotide primers V_1_ (5′AAAACGCCAGCAACGCGGCCT) and V_2_ (5′AGGGCGAATTCCAGCACA), and topo IV cleavage assays allowed mapping to a 50- to 100-bp region. Use of a third oligo V_3_ (5′ GTATTGGGCGCTCTTCCGCT) in conjunction with primer V_1_ amplified a 195-bp V-site PCR product that was suitable for high-resolution sequence analysis.

Primers V_1_ and V_3_ (10 pmol) were 5′-end-labelled by incubation with [γ-^33^P]ATP (10 pmol) and T4 polynucleotide kinase (5 U) in a total volume of 10 µl. Reaction was conducted at 37° C and then the kinase was heat inactivated at 90°C for 3 min and the DNA was recovered and purified on spin columns. Labelled primers were used in PCR to produce cycle sequencing chain termination ladders and to amplify the 195-bp V-site PCR fragment from pCR 2.1 TOPO uniquely end-labelled for cleavage analysis. To label one or other strand, PCR was carried out with 10 pmol of one 5′-end ^33^P-labelled primer and an equal amount of unlabelled reverse primer (V_1_ and V_3_ extend the top and bottom strands, respectively). DNA fragments were purified on spin columns.

Cleavage of V-site DNA end-labelled in the top or bottom strand (∼0.2 pmol) was carried out at 37°C for 1 h with ParC (0.45 µg) and ParE (1.7 µg) in 35 mM Tris–HCl, pH 7.5, 6 mM MgCl_2_, 24 mM KCl, 5 mM DTT, 6.5% glycerol and 50 µg/ml BSA. Gemifloxacin was included at 10 µM. Reactions were stopped by addition of SDS to 1%, and after incubation with proteinase K, DNA products were precipitated with ethanol. Samples were resuspended in 40 µl of loading buffer (95% formamide, 10 mM NaOH, 0.25% bromophenol blue, 0.25% xylene cyanol), heated at 90°C for 2–4 min and separated by electrophoresis in an 8% denaturing urea-polyacrylamide gel. Gels were fixed in 10% v/v acetic acid and 10% v/v methanol, dried under vacuum on to Whatman 3MM filter paper, exposed to a PhosphorImager screen and visualized using software supplied by Amersham Biosciences and ImageQuant. The position of the cleavage site on the labelled DNA strand was determined by comparison of cleavage fragment mobility with a dideoxy sequencing ladder run alongside that had been generated by V_1_- and V_3_-labelled primer extension (using the fmol® DNA cycle sequencing kit) and the same labelled primer utilized in making the end-labelled DNA substrate.

A similar approach was adopted to determine the sites of cleavage at mutant E-sites using 256-bp PCR products labelled on one strand by using 5′-end ^33^P-labelled E-FOR and unlabelled E-REV primers.

### Inhibition of pBR322 cleavage by short duplex DNAs

Wild-type and mutant E-sites were tested for their ability to act as inhibitors of topo IV cleavage of supercoiled plasmid pBR322. The wt E-site duplex was generated by annealing highly purified complementary 34-mer oligonucleotides: 5′-ACCAAGGTCATGAATGACTATGCACG TAA AACAG and 5′-CTGTTTTACGTGCATAGTCATTCATGACCTTGGT. Mutant E-sites M2C, M4C and M1A were produced from complementary oligonucleotides bearing the appropriate symmetrical base changes in the gate region (underlined). In the annealing protocol, 2.5 nmol of each oligonucleotide in reaction buffer (50 µl) containing 10 mM Tris–HCl, 10 mM MgCl_2_ and 50 mM NaCl was heated at 65°C for 30 min in a PCR machine and then cooled slowly (2°C per min) to room temperature. The concentration of each duplex DNA was determined by Nanodrop before inclusion in a standard cleavage assay containing supercoiled pBR322 (400 ng), topo IV and 0.17 µM gemifloxacin. Cleavage products were separated and analysed by electrophoresis in a 1% agarose gel.

## RESULTS

### Functional determinants of quinolone-promoted topo IV-DNA cleavage revealed by a novel competitive assay

**Figure 2.**
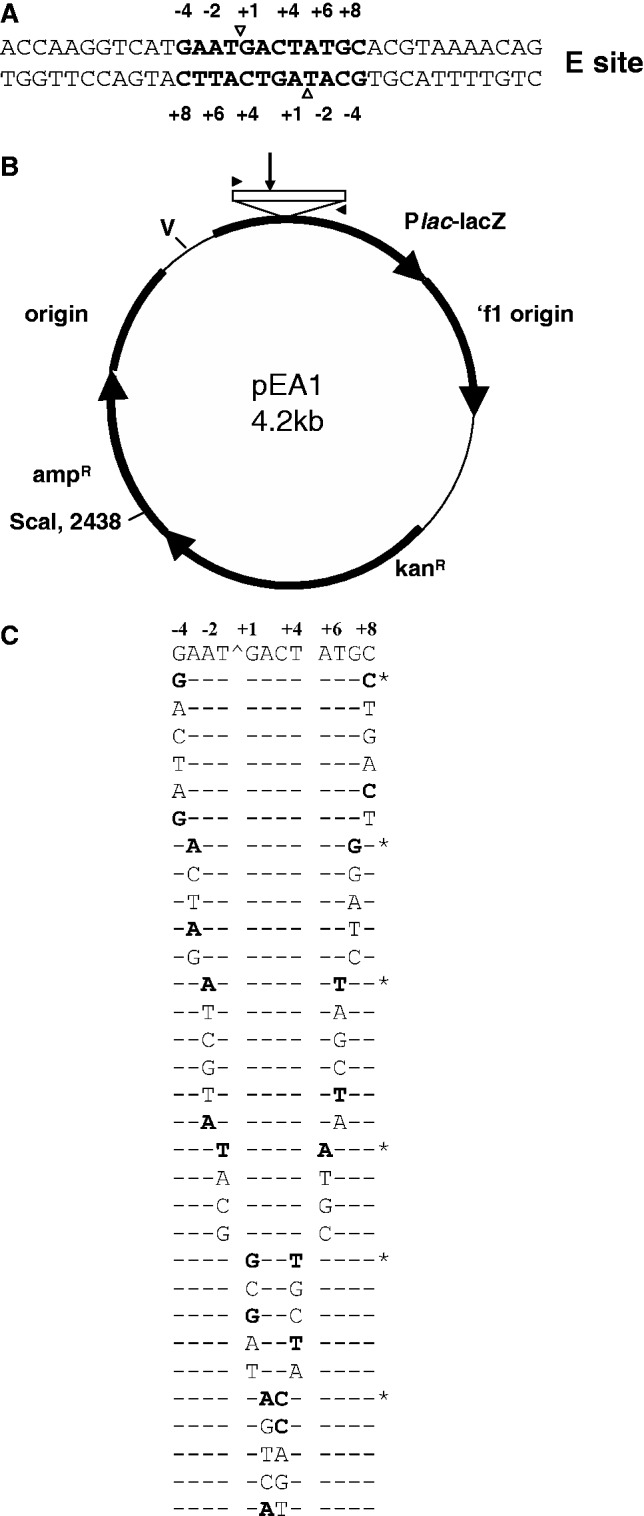
Structure (**A**), cloning (**B**) and mutagenesis (**C**) of the E-site. (A) DNA sequence of the 34-bp E-site that is strongly cleaved by *S. pneumoniae* topo IV in the presence of gemifloxacin. DNA scission to generate a staggered double-stranded DNA break occurs at positions shown by open arrowheads, i.e. between the −1 and +1 nt on each strand within a 12-bp sequence bearing consensus preferences and targeted here for mutagenesis. (B) Cloning of the E-site to generate plasmid pEA1. The site contained in a 256-bp PCR fragment (open box) was inserted directly by topo cloning into vector pCR2.1 TOPO. Filled arrowheads denote oligonucleotide primers used to amplify the E-site PCR fragment. V denotes the ‘V-site’, a second location of topo IV cleavage reported in this study. (C) Mutant E-sites used in this study. The changes indicated were introduced at the cloned E-site of plasmid pEA1 by oligonucleotide-directed mutagenesis. The wt E-site sequence and wt bases are denoted by an asterisk and bold capitals, respectively.

**Figure 3.**
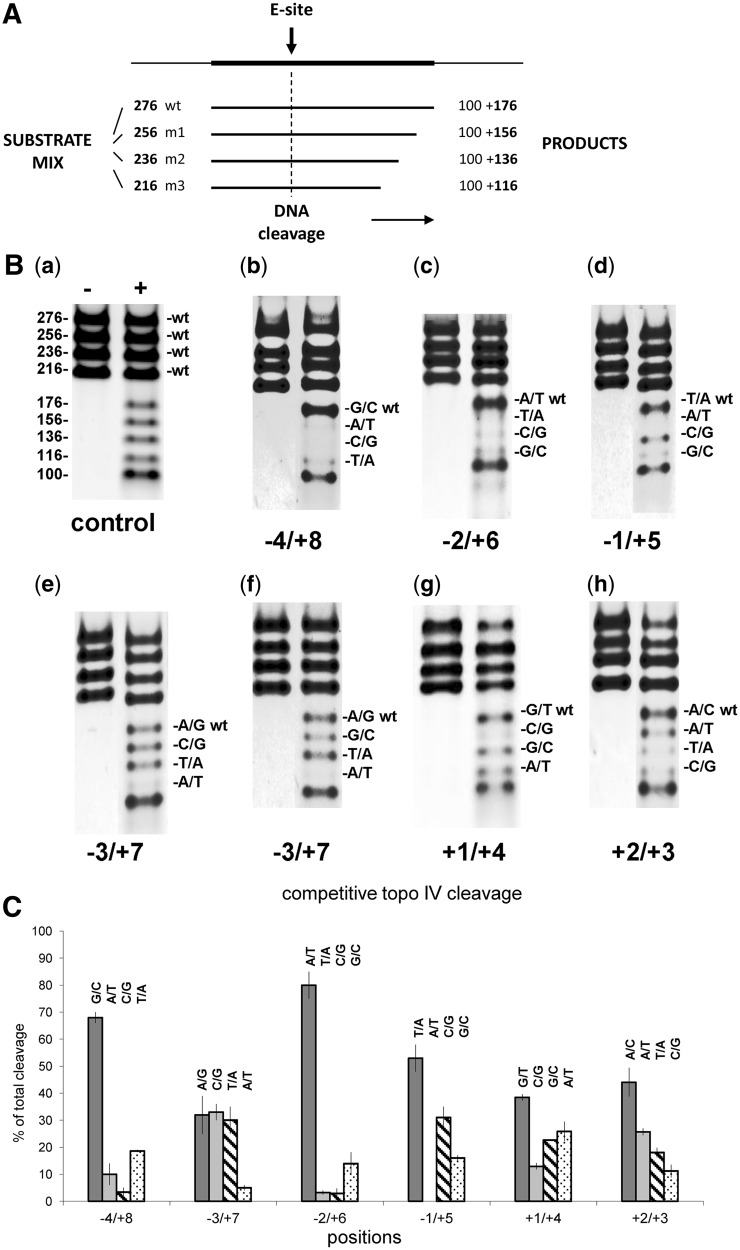
Analysis of DNA breakage at wt and mutant E-sites using a novel competitive cleavage assay. (**A**) Schematic diagram showing the basis of the assay. The wt and mutant (m1, m2 or m3) E-sites are amplified by PCR from plasmid pEA1 and its mutant variants to generate 276-, 256-, 236- and 216-bp fragments, respectively. Equal amounts of each of the four substrates are mixed and incubated with topo IV producing signature 176-, 156-, 136- and 116-bp cleavage fragments and a 100-bp product common to all sites. The four substrate and five cleavage fragments are separated by electrophoresis allowing the efficiency of cleavage at the mutant sites to be compared directly with wt. (**B**) A representative assay for gemifloxacin-promoted cleavage by topo IV. Panel (a), all four cleavage substrates (0.1 µg each) carried a wt E-site generating well-separated 176-, 156-, 136- and 116-bp products. Panels (**b–h**) each used a mix of wt E-site substrate (276-bp) with three mutant site substrates (256-, 236- and 216-bp) carrying changes at −4/+8 (panel (b)), −2/+6 (c), −1/+5 (d), −3/+7 (e and f), +1/+4 (g) and +2/+3 (h). All reactions contained topo IV and 6 mM MgCl_2_ and were carried out in the absence (±) or presence (+) of 320 µM gemifloxacin. Substrates and products were separated by electrophoresis in 4–12% gradient polyacrylamide gels and DNA bands were stained and photographed. Cleavage at each mutant site compared with the internal E-site control is indicated by the relative amounts of the cleavage products. Reduced fluorescence of the smaller cleavage products relative to the wt 176-bp band is largely compensated by the relatively greater number of sites presented per microgram of the smaller substrates. (C) Quantitation of cleavage products from wt and mutant E-sites in the competitive assay. Dark grey bar denotes the wt product. Data represent the average of two independent experiments: bars are standard error of the mean.

To investigate the biochemical requirements for DNA cleavage by bacterial type II topoisomerases, we used the E-site present in the *S. pneumoniae parE* gene (23). It is the strongest topo IV site within an 8-kb region of the pneumococcal chromosome and displays a high degree of 2-fold symmetry with −4G, −2A and −1T present on each strand corresponding to +8C, +6T and +5A on the complementary strand (Figure 2A). The E-site was amplified as a 256-bp PCR fragment and cloned into vector pCR2.1 TOPO to yield plasmid pEA1 (Figure 2B), which was used as a template in oligonucleotide-directed mutagenesis to introduce a variety of symmetric pairwise and single mutations into the −4 to +8 region (Figure 2C). The mutant plasmids were used to develop a new competitive assay in which wt and mutant E-sites (m1, m2 and m3) contained, respectively, on 276-, 256-, 236- and 216-bp PCR products (amplified from pEA1 and its mutants), are mixed in equal weight, cleaved in parallel by topo IV in the presence of fluoroquinolone and the signature 176-, 156-, 136- and 116-bp cleavage products (plus the 100-bp partner fragment) are separated and detected by gradient polyacrylamide gel electrophoresis (Figure 3A). By providing a choice of independent substrates, the assay compares breakage at mutant sites with that of the wt site internal control and requires only a few gel lanes to display the full cleavage repertoire.

Figure 3B displays representative results for topo IV cleavage promoted by gemifloxacin. Panel (a) shows a control experiment in which four starting substrates each bearing the wt E-site were mixed with topo IV in the absence (−) or presence (+) of drug, and cleavage was revealed by denaturation with SDS. Following incubation with proteinase K (to remove covalently bound ParC), DNA products were separated and visualized. The gel system nicely resolves all four DNA substrates as well as the expected 176-, 156-, 136-, 116- and 100-bp cleavage products. Appropriate mixes of wt 276-bp substrate and mutant E-site substrates were used in panels (b)-(h) to examine the effects of mutations at each position in the −4 to +8 region. Cleavage by topo IV was detected only in the presence of gemifloxacin (cf – and + lanes in each panel). From panels (b)-(d), it is clear that any pairwise change of the symmetric wt −4G/+8C, −2A/+6T and −1T/+5A nucleotides resulted in the absence or marked reduction in the yield of signature cleavage products: in each case, only the wt E-site was strongly cleaved, producing the prominent 176-bp product. By contrast, all base combinations at −3/+7 positions produced wt levels of cleavage product except A/T for which the yield of 116-bp product was drastically reduced (panels (e) and (f)). Similarly for +1/+4 positions, purine/pyrimidine pairs G/T, G/C and A/T favoured efficient cleavage, whereas C/G blocked cleavage as shown in panel (g). Lastly, panel (h) indicated selectivity at the +2/+3 positions with the A/C and A/T combinations favoured, and T/A and C/G disfavoured. Inclusion of ATP (at 1 mM) did not affect outcomes (results not shown).

Product bands from two independent sets of competitive cleavage experiments involving 20 mutant sites covering −4 to +8 positions were scanned and quantitated, allowing cleavage at the mutant sites to be compared directly with that of the wt E-site present as internal control (Table 1). The Table additionally includes data for the +2/+3 positions not shown in Figure 3B, revealing cleavage of the +2 G/+3 C mutant site. Representative data for each base position are presented in Figure 3C. Scrutiny of each position highlights the stringent base requirements at −4, −2 and −1 but greater sequence tolerance at −3, +1 and +2 positions. Interestingly, most mutations of the E-site sequence inhibited cleavage, suggesting it is a highly preferred site. Clearly, the new assay reveals a complex set of functional requirements for optimal drug-promoted cleavage at the gate.

**Table 1.** Quantitation of cleavage products generated in competitive assays by quinolone-mediated DNA breakage by topo IV

Position	E-site	Percent of total cleavage		
		exp 1	exp 2	mean
**−4/+8**	G/C*	**66**	**70**	**68**
	A/T	14	6	10
	C/G	1.8	5	3.4
	T/A	18.2	19	18.6
−**3/+7**	A/G*	**39**	**25**	**32**
	C/G	30	36	33
	T/A	25	35	30
	A/T	6	4	5
	A/G*	**37.5**	**37.7**	**37.6**
	G/C	25.3	24.3	24.8
	T/A	24.9	24.7	24.8
	A/T	12.3	13.3	12.8
−**2/+6**	A/T*	**75**	**85**	**80**
	T/A	2.4	4	3.2
	C/G	4.5	1.3	2.9
	G/C	18.1	9.7	13.9
−**1/+5**	T/A*	**58**	**48**	**53**
	A/T	0	0	0
	C/G	27	35	31
	G/C	15	17	16
**+1/+4**	G/T*	**39.3**	**37.6**	**38.45**
	C/G	13.8	12.1	12.95
	G/C	22.6	22.7	22.65
	A/T	24.2	27.5	25.85
**+2/+3**	A/C*	**40.3**	**47.8**	**44.05**
	A/T	26.6	24.8	25.7
	T/A	19.3	16.8	18.05
	C/G	13.8	10.47	12.1
	A/C*	**54**	**42**	**48**
	G/C	21	35	28
	T/A	10	6	8
	C/G	15	17	16

Asterisks denote cleavage of wt E-site; exp 1, exp 2 indicate independent experiments. Bold values are data for the wt E-site.

### Topo IV and gyrase cleavage by single substrate assay

The competitive assay is novel and therefore gemifloxacin-induced E-site cleavage was checked using the conventional approach in which single substrates (256-bp PCR products) are cleaved in separate reactions (Supplementary Figures S1 and S2). Essentially the same functional rules were uncovered for topo IV although subtle preferences, e.g. at +1/+4 and +2/+3 were less obvious under the forcing conditions of the single substrate assay (Supplementary Figure S1). In addition, the +1 T/+4 A site (not tested in competitive assays) was not cleaved by topo IV (results not shown). For gyrase, weaker E-site cleavage precluded competitive analysis. However, single substrate studies revealed that the functional rules for gyrase overlapped those of topo IV (shared −4G and −2A) but differed in unique preferences (for −3T and −1T) and degenerate preferences at +2/+3 (Supplementary Figure S2). Overall, the competitive assay was less laborious and easier to quantitate.

### Intrinsic versus drug-specific determinants

**Figure 4.**
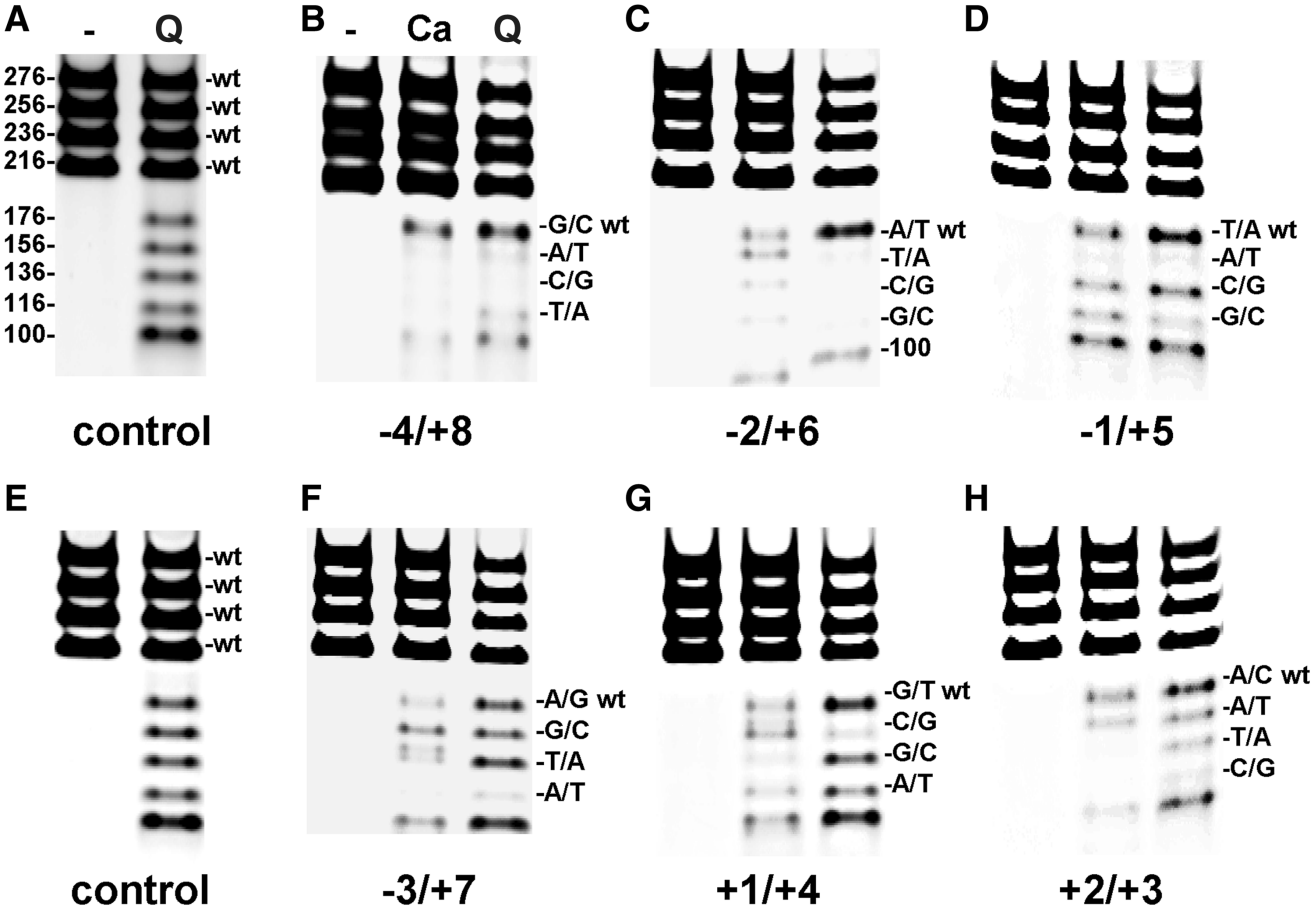
Comparison of Ca^2+^- and quinolone-mediated cleavage specificity by topo IV using the competitive assay. Mutant E-sites were mixed with the wt site in equal amount (0.1 µg, determined by Nanodrop) and cleaved with topo IV in the absence (−) or presence of 32 mM Ca^2+^ (Ca) or 320 µM gemifloxacin plus 6 mM MgCl_2_ (Q). Reaction products were separated and analysed on 4–12% polyacrylamide gradient TBE gels as described in the Figure 3 legend. Panels (**A** and **E**) show the control gemifloxacin cleavage reactions in which the 276-, 256-, 236- and 216-bp substrates each carried a wt E-site generating 176-, 156-, 136- and 116-bp products plus the common 100-bp partner. Drug-induced cleavage at the E-site was 2- to 3-fold more efficient than the Ca^2+^-mediated reaction, and therefore to facilitate comparison, only half of the reaction products from each gemifloxacin assay was loaded for comparison in Panels (**B–D**, **F–H**).

It is known that Ca^2+^ induces topo IV-DNA breakage at the same sites as seen with Mg^2+^ (15) (though more efficiently). Therefore, a complete mutational analysis of cleavage in the presence of Ca^2+^ should yield information on the ‘intrinsic recognition’ of a DNA cleavage site by topo IV. In principle, comparison with quinolone data would then distinguish enzyme-specific and drug-dependent contributions to DNA cleavage selectivity. Figure 4 presents a competitive assay of Ca^2+^-promoted DNA breakage by topo IV at the E-site and its mutants (lanes Ca) with the quinolone-reactions run alongside (lanes Q) [To confirm the results, single substrate assays (Supplementary Figure S3) were carried out in parallel]. It is evident that nearly all the mutations at −4 to +8 positions that block drug-induced cleavage also inhibited Ca^2+^ cleavage (panels B, C, D and F), indicating that, for most positions, the enzyme plays the key role in cleavage site selectivity. In particular, the results suggest that sequence preferences at −4/+8, −3/+7, −2/+6 and −1/+5 are determined primarily by the enzyme, whereas the drug is a major determinant of preferences at +1/+4 and +2/+3. Thus, Ca^2+^- and quinolone-cleavage profiles were similar for mutants at −4/+8 (panel B), −1/+5 (panel D) and at −3/+7 (though partial cleavage of the −3 T/+7 A substrate at an alternative site led to an additional product band) (panel F). Similarly, E-site mutations at −2A/+6T block both drug and Ca^2+^ cleavage, in the latter case revealing cleavage at an alternative site and producing the differently sized fragments seen in Figure 3, panel C and Supplementary Figure S3. Subtle differences in Ca^2+^ versus quinolone cleavage preferences observed at −4 to −1 positions may arise from known changes in helix conformation of the DNA gate occasioned by drug binding (28). By contrast, enzyme- and drug-mediated cleavage preferences were different at both +1/+4 and +2/+3 with +1C/+4G most favoured in the Ca^2+^-promoted reaction but strongly disfavoured for gemifloxacin cleavage (Figure 4, panels G and H, Supplementary Figure S5). The results show unequivocally that the drug can override enzyme preferences to confer the different +1/+4 and +2/+3 base preferences seen in quinolone-promoted cleavage.

### Drug sensing of the cleaved DNA gate

**Figure 5.**
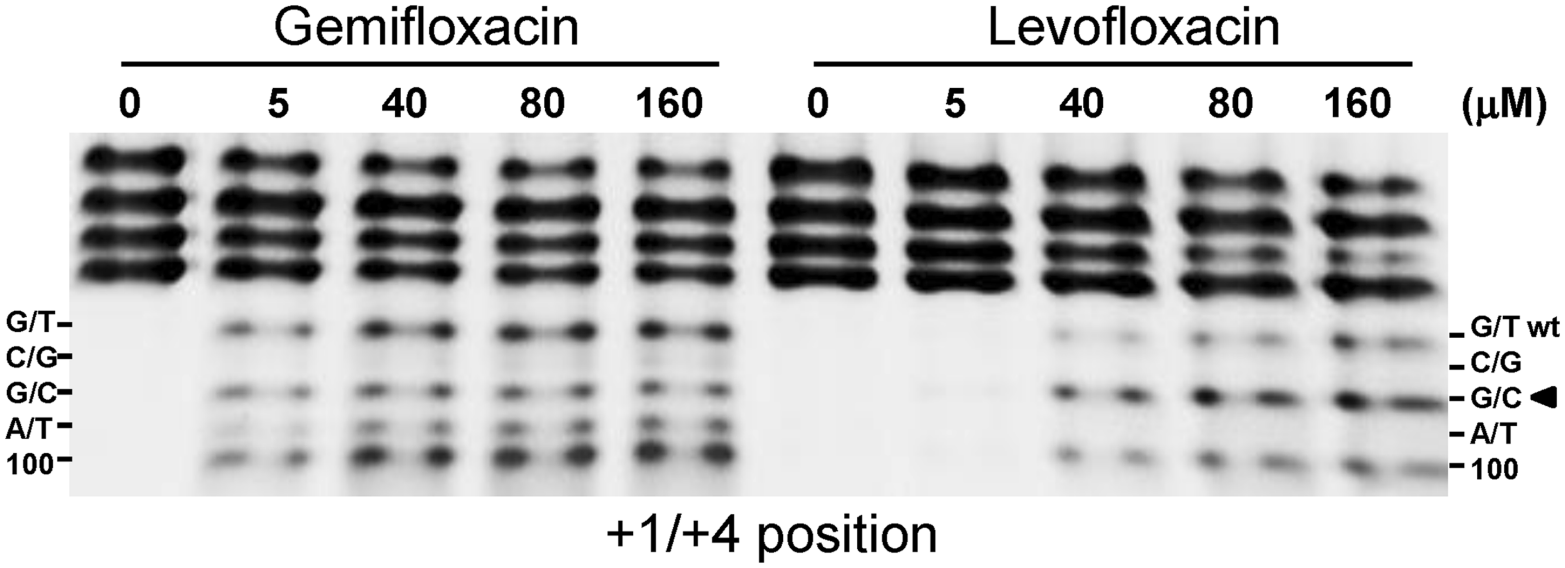
Drug structure-specific preferences at +1. Symmetric +1/+4 mutant E-sites were mixed with the wt site (+1G/+4T), cleaved with topo IV in the presence of gemifloxacin and levofloxacin at the concentrations shown, and the products were separated and analysed on 4–12% polyacrylamide gradient TBE gels as described in the Figure 3 legend. A 100-bp cleavage product is generated by breakage at any cleavable E-site.

The drug-mediated preference for purines at +1 positions (Figure 3 and 4) is interesting given that recent X-ray crystal structures of the topo IV–E-site cleavage complex reveal two quinolone molecules stacked against the +1 bases (27,28). To examine the sequence preferences of different quinolones, we compared topo IV cleavage of linear pEA1 plasmid mediated by six fluoroquinolones currently in clinical use: the antipneumococcal drugs gemifloxacin, trovafloxacin, moxifloxacin, levofloxacin and sparfloxacin, plus ciprofloxacin used against Gram-negative pathogens (Supplementary Figure S4). All six quinolones promoted cleavage at a similar spectrum of sites including the E-site but with efficiencies determined by the drug. We selected the structurally distinct gemifloxacin and levofloxacin (Figure 1) for more detailed comparison using the competitive assay and the −4, −2 and +1 E-site mutants used in Figure 3B. Levofloxacin is less potent than gemifloxacin (32) and therefore a range of different drug concentrations was used to facilitate comparison at similar levels of cleavage. Levofloxacin recapitulated the −4 and −2 preferences seen in Figure 3 for gemifloxacin (results not shown). However, whereas gemifloxacin was able to promote cleavage of E-sites with a variety of purine/pyrimidine combinations at +1/+4, levofloxacin showed a marked preference for the +1G/+4C substrate yielding predominantly the G/C cleavage product rather than the wt (G/T) product and with little or no breakage of C/G and A/T sites (Figure 5). These biochemical results show directly for the first time that cleavage preferences at the +1 positions are modulated by the molecular structure of the quinolone.

### DNA context: a novel plasmid site for topo IV

To investigate the generality of sequence preferences, we sought to extend cleavage studies to a second site derived from a different biological and sequence context. By chance, cleavage of the full-length pEA1 plasmid (rather than insert PCR products) revealed strong topo IV cleavage at a second site that mapped to just outside the replication origin region of the pCR2.1 TOPO vector and which we termed the ‘V’ or vector site (Supplementary Figure S4, Figure 2B). PCR products containing the V-site and 5′-^33^P-labelled on one or other DNA strand were generated from pEA1 DNA, cleaved by topo IV in the presence of gemifloxacin, and DNA breakage mapped at nucleotide levels by comigration on high-resolution DNA sequencing gels against a ladder of dideoxy chain termination products generated for the same PCR fragment (results not shown). Cleavage occurred uniquely between vector pCR2.1 TOPO nucleotides 3841T and 3842A (+1 position asterisked) in the sequence.

5′ GGAT *AACC GTAT, which is also present in pBR322 (nt 2431) and other ColE1/pMB1-based vectors. Despite its different provenance, the V-site is closely homologous to the E-site, retaining the important −2A/+6T bases, the presence of purine bases at +1 positions, A and C bases at +2 and +3 positions and half-site conservation of preferred −4G and −1T. Discovery of the V-site allowed functional determinants to be examined in a different sequence background.

### Shared determinants in cleavage at E- and V-sites

**Figure 6.**
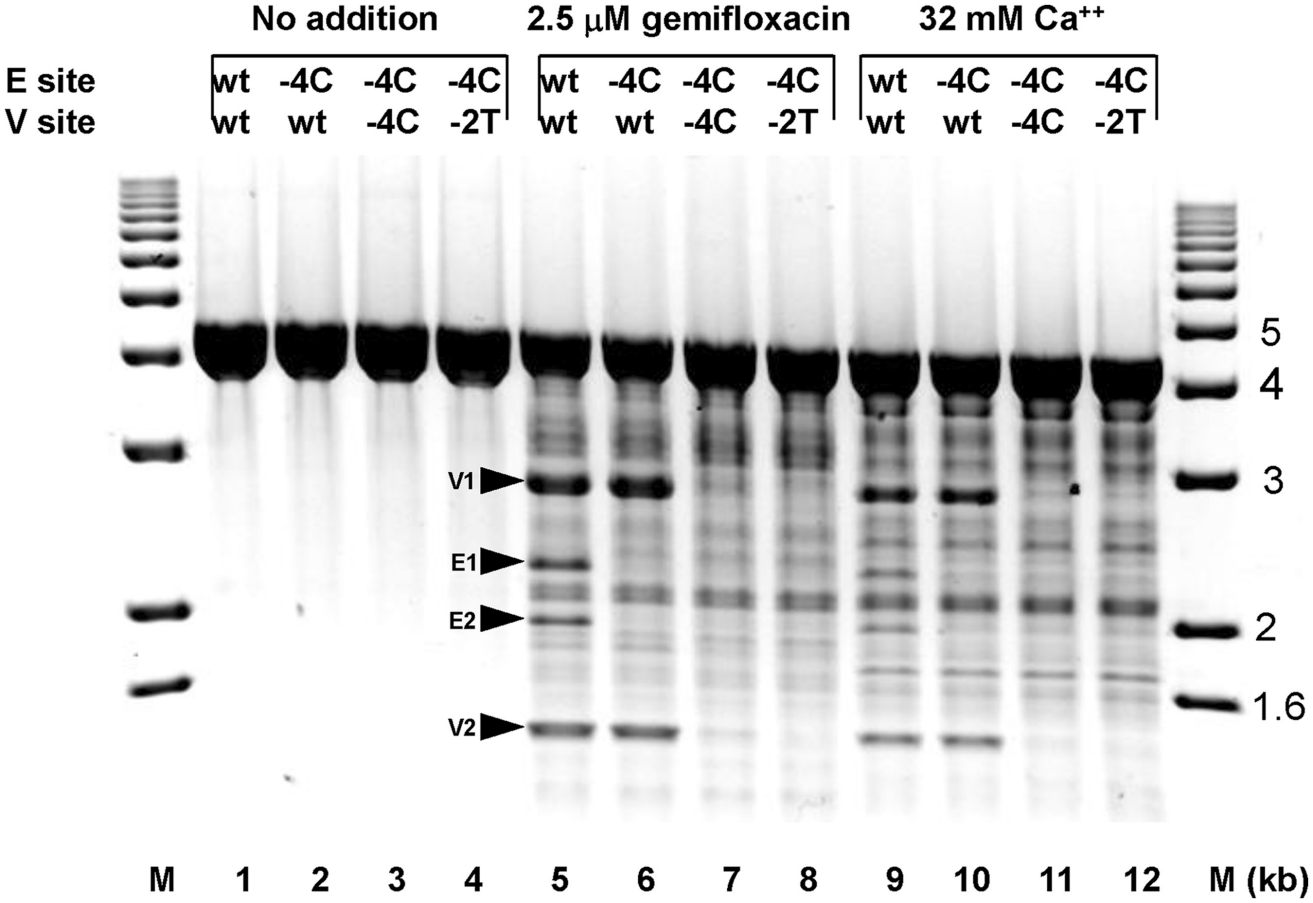
Gate mutations at E- and V-sites inhibit topo IV cleavage. ScaI-linearized plasmid pEA1 bearing wt or mutant E- and V-sites was incubated with topo IV alone (lanes 1–4) or in the presence of 2.5 µM gemifloxacin (lanes 5–8) or 32 mM Ca^2+^ (lanes 9–12). DNA cleavage was induced as described in previous figure legends and DNA products were examined by electrophoresis in a 1% agarose gel. Cleavage fragments V1 and V2, and E1 and E2, arising from breakage at the V- and E-sites are indicated by arrows. The presence of wt or mutant sites is indicated above each lane: −4C and −2T denote the presence of pairwise −4C/+8G or −2T/+6T mutations, respectively. M, DNA size markers.

To compare topo IV action at E- and V-sites, we used linear pEA1 plasmid substrates bearing mutations at one or both sites in the presence of either 6 mM MgCl_2_ and gemifloxacin, or 32 mM Ca^2+^ ions (Figure 6). In the absence of drug or Ca^2+^, there was no topo IV-induced plasmid breakage (lanes 1–4). Inclusion of gemifloxacin promoted cleavage at a spectrum of sites, and as expected substitution of guanines at −4 with disfavoured cytosines abrogated gemifloxacin-stimulated cleavage at the E-site eliminating the E1 and E2 products (Figure 6, cf lanes 5 and 6). Introduction of −4C mutations or −2T changes on both strands of the V-site blocked cleavage at the site indicated by the absence of V1 and V2 products (cf lanes 5, 7 and 8). Calcium ions (substituted for quinolone and Mg^2+^) also induced plasmid cleavage by topo IV but with differences in the spectrum of sites compared with that seen with gemifloxacin (Figure 5, cf lanes 5 and 9). E- and V-sites were both cleaved in the presence of Ca^2+^ and the same mutations at −2 and −4 positions that blocked drug-dependent DNA breakage (lanes 5–8) also inhibited Ca^2+^-promoted DNA scission (lanes 9–12). Blockade was not reversed by using supercoiled pEA1 as substrate (results not shown). Similar to quinolone cleavage, Ca^2+^ cleavage reactions were reversed by EDTA, salt and heat (results not shown). Overall, the results indicate that key determinants of quinolone and Ca^2+^ cleavage uncovered at the E-site also apply to the V-site validating the functional rules (Figure 3).

### Non-cleavable E-sites bind topo IV

**Figure 7.**
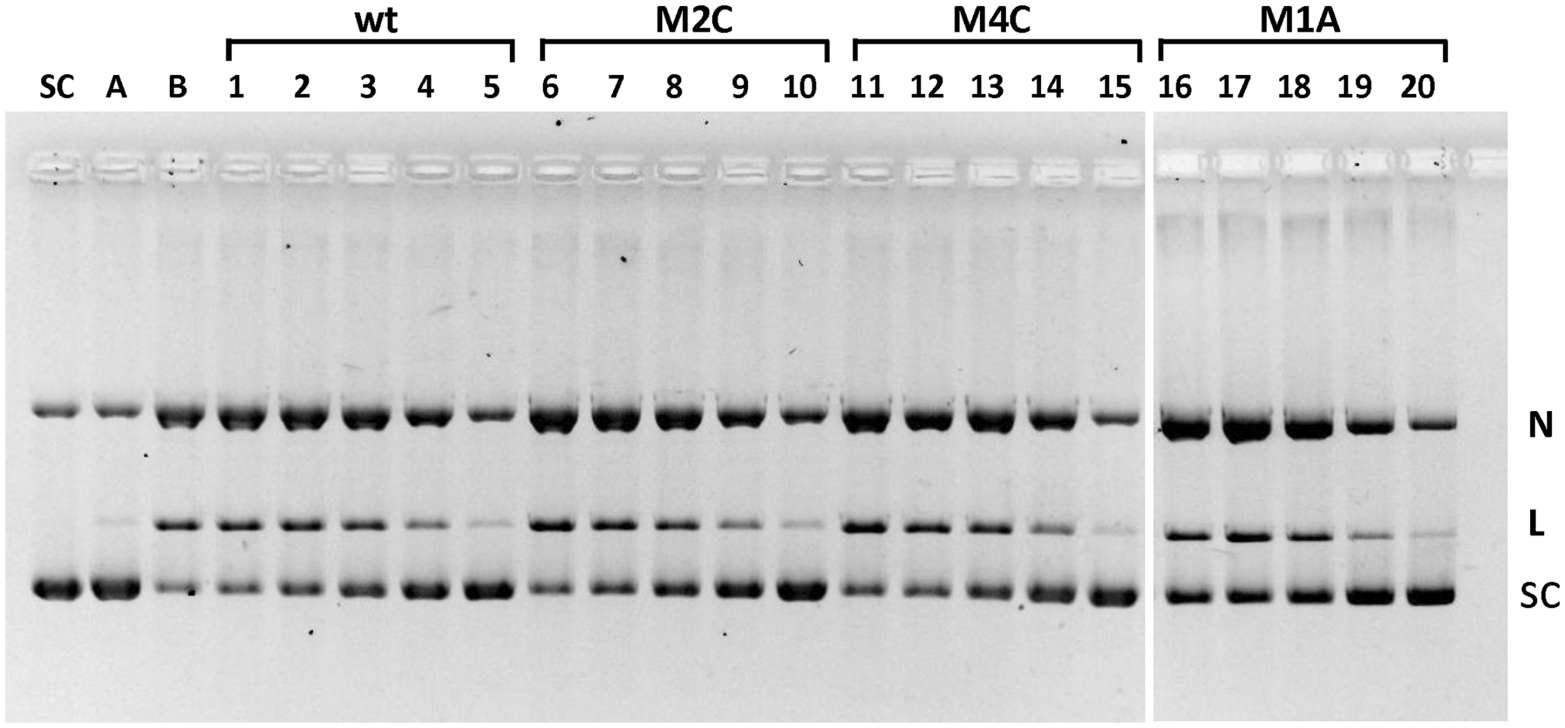
Mutant E-sites bind and inhibit topo IV. Supercoiled pBR322 DNA (400 ng) was incubated with topo IV and 0.17 µM gemifloxacin in the absence (lane B) or presence of 34-mer DNA duplexes comprising the E-site sequence (wt) or mutant sites M2C, M4C or M1A bearing −2C/+6G, −4C/+8G and −1A/+5T alterations, respectively (lanes 1–20). For each set of lanes 1–5, 6–10, 11–15 and 16–20, the concentrations of 34-mer were 1.5, 3, 6, 12 and 30 µM, respectively. Cleavage conditions were as described in Figure 3. After SDS and proteinase K treatment, plasmid DNA products were separated and displayed by gel electrophoresis in 1% agarose. Lane SC, supercoiled pBR322 substrate; lane A, as lane B but omitting gemifloxacin. N, L and SC denote nicked, linear and supercoiled plasmid bands.

It is remarkable that many mutant E-sites were refractory to DNA cleavage even at high drug concentrations (Figure 3). To examine whether cleavage inhibiting mutations act to prevent binding of topo IV to its 34-bp binding site on DNA (33), we tested short 34-mer oligonucleotide duplexes comprising wt or mutant E-sites as inhibitors of topo IV-mediated cleavage of plasmid pBR322 (Figure 7). Cleavage of supercoiled pBR322 was efficient even at low gemifloxacin levels (0.17 µM), producing linear and nicked DNA (Figure 7, lane B), reflecting the 16-fold greater affinity of topo IV for supercoiled DNA over linear DNA (33) and the inherently greater sensitivity whereby each cleavage event produces linear DNA. Inclusion of increasing amounts of wt E-site duplex decreased topo IV cleavage in a dose-dependent manner (lanes 1–5) with almost complete inhibition at 30 µM (lane 5). Mutant E-sites M2C, M4C or M1A, intrinsically resistant to cleavage through −2C/+6G, −4C/+8G or −1A/+5T mutations (Figure 3C), all inhibited pBR322 cleavage with essentially the same efficiency as the wt site yielding an estimated IC_50_ of 6–12 µM (lanes 6–20). Evidently, the mutant sites bind topo IV as efficiently as the wt E-site, a result that has important mechanistic implications.

## DISCUSSION

We have established the functional determinants governing DNA cleavage by topo IV and gyrase, the first comprehensive analysis for these bacterial type II topoisomerases. By site-directed mutagenesis of the chromosomal E-site and of the plasmid V-site, we defined the overlapping but distinct sequence requirements for quinolone-promoted cleavage by gyrase and topo IV, the features of an ‘optimal site’ and the contributions of enzyme and drug to site selectivity defined through parallel studies of Ca^2+^-promoted DNA breakage. Using these results and insights from X-ray crystal structures, we suggest a plausible model for DNA selectivity by type II enzymes.

Previous studies of topo IV and gyrase have provided only limited biochemical data on quinolone-promoted DNA cleavage specificity (20,21,33). We used systematic mutagenesis to investigate breakage at the E-site (Figure 2) chosen for its strong cleavage by *S. pneumoniae* topo IV (and, less efficiently, by gyrase) (23) and for which we have determined recent X-ray crystal structures of topo IV–E-site cleavage complexes (27,28). To examine up to 25 mutant sites, we developed a novel cleavage assay in which wt and mutant E-sites are mixed together, cleaved and the signature products are separated and analysed by gel electrophoresis. The method provides a simple visual display of base selectivity at each position (Figure 3). For gemifloxacin-induced DNA cleavage by topo IV, any pairwise mutation of the symmetric −4G/+8C, −2A/+6T and −1T/+5A abrogated or reduced DNA scission (as did asymmetric single mutations at −4 or −2 positions, Supplementary Figure S5). There was also selectivity at −3, +1 and +2 positions with the −3 positions showing the greatest sequence tolerance. Strictly speaking, the results relate to the E-site but we note the validity of key preferences was confirmed in the different sequence context of the V-site (Figure 6). Assuming position-specific preferences act independently, the data define a set of functional requirements for gemifloxacin-promoted cleavage by topo IV, namely **G**(N/a)**A**(T/a)*(Pu/py)(A/c)(C/g)(Py/pu) (A/t)**T**(N/t)**C** (strongly preferred bases in bold capitals; preferred bases in capitals; strongly disfavoured bases are in lower case font; N indicates no strong preference; Pu, purine; Py, pyrimidine; asterisk indicates DNA scission between −1 and +1 bases). These functional rules differ from those of gyrase 5′**G**T**AT** *cNNg **AT**A**C** (Supplementary Figure S2) [consensus GN_4_G(G/c)(A/c)G *GNNCtTN(C/a) (23)] and contrast with the simple recognition sequences of many restriction enzymes, perhaps reflecting the inherently greater complexity of operating and arresting a reversible DNA gate.

Several important insights emerge from the mutational analysis. First, the functional rules for gemifloxacin-induced breakage were much more stringent and showed key differences compared with the topo IV consensus G(G/c)(A/t)a*GNNCt(T/a)N(C/a) determined on pneumococcal DNA using gemifloxacin (23). Whereas statistically favoured or disfavoured bases at the −4, −2 and −1 positions correctly predicted outcomes, surprisingly sites with ‘neutral’ bases were also poorly cleaved viz A/T, C/G at −4/+8, −2G/+6C, −1G/+5C and most combinations at +2/+3 (Table 1, Figure 3C). There also were differences in −3/+7 outcomes. Perhaps through sequence bias or inclusion of weak sites, consensus data need not predict biochemical outcome. Second, a variety of single mutations blocked cleavage, suggesting many individual elements are crucial for DNA scission (Figure 3). Third, the quinolone contributes to DNA selectivity (Figure 4 and 5). Fourth, the E sequence is highly optimized for cleavage, most mutations reduced cleavage (Figure 3). Interestingly, loss of E- and V-sites did not affect plasmid recovery or topology maybe due to compensation by other sites (results not shown).

**Figure 8.**
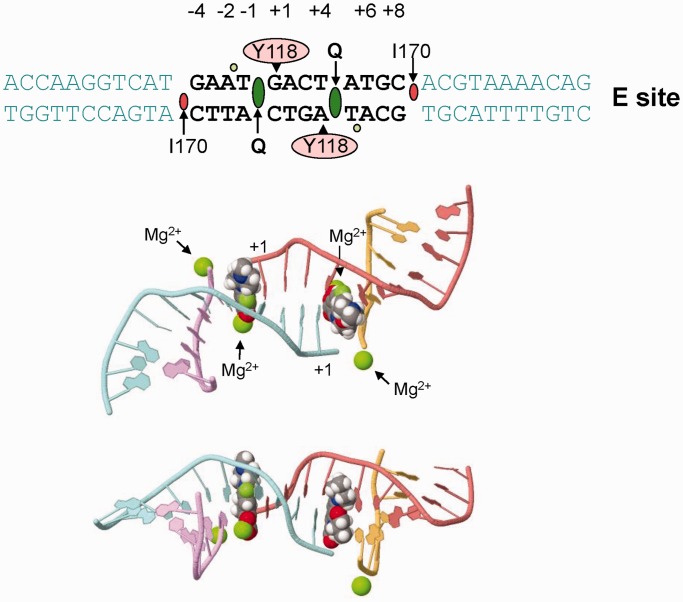
A. DNA bending and molecular interactions in the quinolone–E-site cleavage complex of *S. pneumoniae* topo IV. Top panel shows the 34-bp E-site DNA sequence highlighting the key positions engaged in topo IV-DNA and levofloxacin-DNA interactions. Bases in the −4 to +8 region are shown in bold. Active site ParC tyrosines that form a covalent 5′ phosphate link at +1 are shown in pink, intercalating quinolone–Mg^2+^ complexes (Q) and ParC isoleucine side chains are in dark green and red, respectively, and Mg^2+^ ions are shown in light green. Centre and bottom panels depict top and side views of the E-site DNA present in the levofloxacin–topo IV cleavage complex showing the disposition of drug molecules and DNA- and drug-chelated Mg^2+^ ions (centre) within the highly bent DNA. The smaller darker green sphere on each fluoroquinolone molecule corresponds to fluorine. Images were generated with First Glance in JMol based on protein data bank entry 3RAE.

Analysis of Ca^2+^-promoted cleavage by topo IV allowed us to establish the determinants for intrinsic DNA recognition of a cleavage site by topo IV, namely **G(**N**/**a)**A(**N**/**a) *CACG (N/t)**T(**N**/**t)**C** (Figure 4 and Supplementary Figure S3). Comparison with gemifloxacin cleavage indicated that invariant base preferences at −4 to −1 on each strand are conferred primarily by the enzyme, whereas the drug contributes to specificity at +1/+4 and +2/+3 (Figures 4 and 5). Preferred bases map to key interactions in the quinolone-E-site cleavage complex of pneumococcal topo IV (27,28) wherein the gate DNA is sharply bent into a U-shape and cleaved with a drug molecule inserted between −1 and +1 bases on each strand (Figure 8). The bicyclic ring system of the quinolone (Figure 1) is stacked against the tyrosyl-linked +1 base, preventing reversal by distancing the reactive 5′ phosphotyrosine and 3′-OH DNA ends (Figure 8). Quinolones bind poly(dG) > poly(dA) > poly(dT) > poly(dC) (34) with the greater preferences for purines likely accruing through greater base stacking via π–π interactions with the aromatic system of the drug, explaining the +1 purine preference (Figures 3–5). The ParC I170 residues intercalate symmetrically from the minor groove between the +8 and +9 bases on each strand (Figure 8) (28) with the preferred −4G/+8C bases perhaps best accommodating side-chain intercalation to induce DNA bending. There is contact between the −3C of E-site DNA and ParC R28 but no clashes with A at the −3 position on the other strand accounting for the −3 base degeneracy. Surprisingly, no obvious enzyme contacts are seen with the highly preferred −2A/+6T bases (or at +2/+3 positions), but a ParE-chelated Mg^2+^ ion is bound to the non-scissile phosphodiester group linking −1 and −2 nucleotides on each strand, which might be sensitive to the bases present at these positions. Particular preferences at +2/+3 may allow establishment of the pre-cleavage B-A-B helix conformation at the gate (28) or facilitate adjustments needed for drug–DNA intercalation. Evidently, multiple enzyme, DNA, drug and metal ion interactions are involved in stabilizing the bent and cleaved DNA gate.

**Figure 9.**
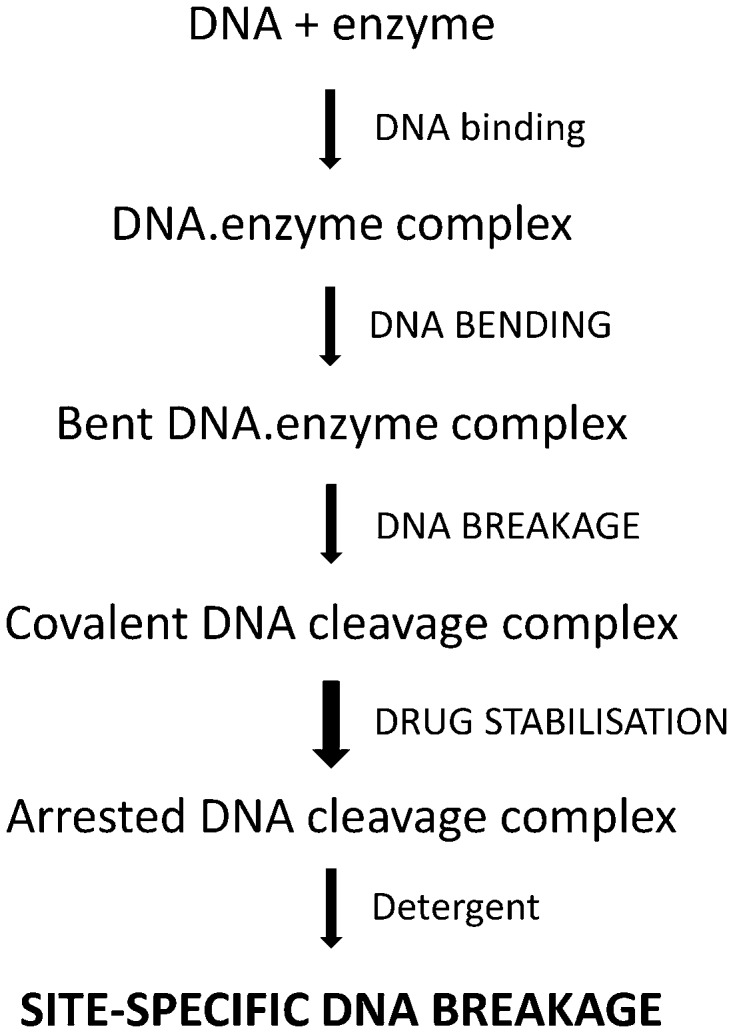
Tentative scheme showing the steps involved in drug-promoted DNA cleavage by a type II topoisomerase. Following non-specific DNA binding to the enzyme, DNA selectivity is exercised at subsequent DNA bending, DNA breakage and/or cleavage stabilization steps. The importance of DNA bending as a prerequisite for DNA cleavage was also noted in a recent article accepted after the present work was submitted (38).

At what stage is DNA selectivity conferred? To approach this issue, we propose a model in which site-specific DNA scission is the end product of sequential enzyme–DNA binding, DNA bending, DNA breakage and drug stabilization (Figure 9). Evidence for this sequence of events comes from X-ray crystal structures of different drug-free E-site complexes with topo IV (28) [and in complexes of gyrase and topo II) (35–37)] in which the DNA is bent and either cleaved or uncleaved, consistent with the idea that enzyme-induced DNA bending precedes DNA cleavage and drug arrest. That E-site mutations did not affect binding to topo IV indicates that DNA discrimination occurs at a subsequent step (Figure 7).

Elegant biophysical studies reported that human topo IIalpha bound two unrelated ‘cleavable’ and ‘uncleavable’ DNA duplexes with similar affinity (39) [(also seen for yeast topo II in (40)]. However, only the cleavable DNA formed a minority high FRET enzyme species suggested to be a bent DNA–enzyme complex. On that basis, DNA sequence discrimination by topo II was attributed to the intrinsic ability of a DNA to undergo bending and distortion before DNA breakage. Were this scenario to apply to topo IV, then the E- and V-sites and their cleavable mutants would constitute a repertoire of DNA sequences capable of bending/distortion, with cleavage-inhibiting DNA mutations acting to block this conformational transition.

Intrinsic DNA bendability could play a role in DNA selectivity but may not be the sole determinant for topo IV with evidence suggesting that ‘enzyme-induced’ DNA bending and drug stabilization are important factors. Thus, the E-site-topo IV structures reveal multiple protein–DNA contacts stabilizing the bent DNA whose alteration through mutation could prevent DNA bending and subsequent cleavage. Indeed, enzyme specific determinants (Figure 4) that differ for topo IV and gyrase (Figure 3 and Supplementary Figure S2) suggest that enzyme contacts are important. Failure to form the bent-DNA enzyme precursor could account for the mutational blockade of both drug- and calcium-mediated cleavage (Figure 4). Secondly, evidence from this work (Figures 4 and 5) and elsewhere indicates the drug itself contributes to DNA discrimination through preferred interactions at −1 and/or +1 positions (22,25,41). Presumably, the enzyme binds non-specifically to DNA, and the drug stabilizes a subset of cleavage sites able to accommodate ligand binding allowing their detection as double-strand breaks. Further work will be needed to delineate the exact contributions of DNA bending and drug stabilization in DNA gate selection and thereby understand the roles of topo IV and gyrase as selective drug targets *in vivo* (42–45).

In summary, we have established the first systematic functional rules for sequence-selective DNA cleavage by topo IV and gyrase, identified enzyme, DNA and drug requirements at the gate and proposed a plausible model for sequence discrimination. The work provides significant insights on type II topoisomerase inhibition by current fluoroquinolones and should aid further development of novel topoisomerase-targeting therapeutics.

## SUPPLEMENTARY DATA

Supplementary Data are available at NAR Online.

## FUNDING

Biotechnology and Biological Sciences Research Council, UK [BB/D014484/1, BB/H00405X/1 to L.M.F.]. Funding for open access charge: Biotechnology and Biological Sciences Research Council.

*Conflict of interest statement*. None declared.

## Supplementary Material

Supplementary Data
