# Supplementary material for: Functional determinants of gate-DNA selection and cleavage by bacterial type II topoisomerases
Source: Nucleic Acids Res. 2013 Aug 10;41(20):9411–23. doi: 10.1093/nar/gkt696 (PMC3814380; doi:10.1093/nar/gkt696)
Supplement: Supplementary Data [file supp_41_20_9411__index.html]

Functional determinants of gate-DNA selection and cleavage by bacterial type II topoisomerases — Functional determinants of gate-DNA selection and cleavage by bacterial type II topoisomerases — Supplementary Data 

# Functional determinants of gate-DNA selection and cleavage by bacterial type II topoisomerases

## Supplementary Data

files

**Files in this Data Supplement:**

- Supplementary Data - doc file
- Supplementary Data - tif file
- Supplementary Data - tif file
- Supplementary Data - tif file
- Supplementary Data - tif file
- Supplementary Data - tif file
