# Supplementary material for: Functional determinants of gate-DNA selection and cleavage by bacterial type II topoisomerases
Source: Nucleic Acids Res. 2013 Aug 10;41(20):9411–23. doi: 10.1093/nar/gkt696 (PMC3814380; doi:10.1093/nar/gkt696)
Supplement: Supplementary Data [file supp_gkt696_nar-01034-f-2013-File014.doc]

**SUPPLEMENTARY TEXT:**

Arnoldi,E., Pan,X-S. and Fisher,L.M. Functional determinants of gate DNA selection and cleavage by bacterial type II topoisomerases.

**Figure S1**. Mutations at the E-site inhibit gemifloxacin-mediated DNA breakage by topo IV. Wild-type and mutant E sites were amplified as 256-bp PCR products from plasmid pEA1 and its mutant derivatives. Each purified DNA (0.2 μg, determined by the Nanodrop method) was incubated individually with topo IV ParC (0.45 μg) and ParE (0.85 μg) and 6 mM MgCl_2_ in the absence or presence of gemifloxacin. After SDS and proteinase K treatment, DNA was analysed by electrophoresis in gradient 4-12% polyacrylamide gels. Gels were stained with ethidium bromide or SYBRGreen dye and photographed under uv illumination. Each gel compares cleavage of the wild-type (wt) E site fragment with three mutant fragments bearing the indicated pairwise changes at particular symmetric positions within the -4 to +8 gate-DNA region. The concentrations of gemifloxacin (μM) used in the reactions are indicated above the lanes. Arrows denote the 156- and 100-bp products generated by cleavage at the E site. For those mutant sites that were cleaved, use of appropriate ^33^P -5’-end-labeled substrates and analysis on high resolution sequencing gels confirmed that cleavage occurred at the same nucleotide positions as the E site itself (results not shown).

**Figure S2**. Quinolone-promoted gyrase action at the E-site. Wild-type and mutant E-site PCR products were generated, purified and used as substrates for gyrase-mediated cleavage. The cleavage protocol was identical to that described for topo IV, namely 0.2 μg of DNA substrate with inclusion of GyrA (0.45 μg) and GyrB (0.85 μg) and gemifloxacin at 0, 80 or 320 μM. Product analysis by gel electrophoresis was carried out as described in the Figure S1 legend. E-site cleavage products are denoted by arrows.

**Figure S3**. Effects of E-site mutations on Ca^2+^-mediated DNA cleavage by topo IV using single substrate assays. Wild-type and mutant E sites were amplified as 256-bp PCR products from plasmid pEA1. Each purified DNA (0.2 μg) was incubated with topo IV in the absence (- lanes) or presence of 32 mM CaCl_2_ (+ lanes). After SDS and proteinase K treatment, DNA products were separated and analysed by electrophoresis in gradient 4-12% polyacrylamide gels as described in the legend to Figure S1. Arrows denote the 156- and 100-bp products generated by cleavage at the E site. Bent arrows indicate cleavage at secondary sites revealed by blocking E-site cleavage.

**Figure S4**. A variety of quinolones mediate topo IV cleavage at E- and V-sites. ScaI-linearised pEA1 (400 ng) was incubated with *S. pneumoniae* topo IV in the absence (lane 0) or presence of various quinolones. DNA cleavage was induced by the addition of SDS and proteinase K. DNA products were examined by electrophoresis in a 1% agarose gel. Quinolone concentrations were adjusted to generate similar levels of DNA cleavage as follows: gemifloxacin (Gemi) at 1.25, 2.5 and 5 μM; trovafloxacin (Trova) at 2.5, 5 and 10 μM; moxifloxacin (Moxi) at 20, 40 and 80 μM; levofloxacin (Levo) at 40, 80 and 160 μM; sparfloxacin (Spar) at 40, 80 and 160 μM; and ciprofloxacin (Cip) at 40, 80 and 160 μM. DNA cleavage produced fragments V1 and V2 and E1 and E2 (arrowheads) arising from breakage at V and E sites, respectively. Lane M, DNA size markers.

**Figure S5**. Topo IV cleavage activity at asymmetrically mutated sites. The competitive cleavage assay was used to compare gemifloxacin-promoted DNA breakage at the wt E site (two symmetric -4G bases, two symmetric -2A bases) with substrates in which one or both bases at each position had been substituted with a disfavoured base. Replacement of either -4G base with a disfavoured A base allowed reduced cleavage by topo IV whereas mutation of both -4 bases to A blocked cleavage. Similarly, substitution of either of the -2A bases (but not both) with disfavoured T also allowed cleavage. Thus, single mutations at the key -4 and -2 positions reduced but did not block DNA breakage. Conditions were as described in Figure 3. Gemifloxacin concentrations (μM) are indicated above the lanes.
