# Supplementary figures and images for: Functional determinants of gate-DNA selection and cleavage by bacterial type II topoisomerases
Source: Nucleic Acids Res. 2013 Aug 10;41(20):9411–23. doi: 10.1093/nar/gkt696 (PMC3814380; doi:10.1093/nar/gkt696)

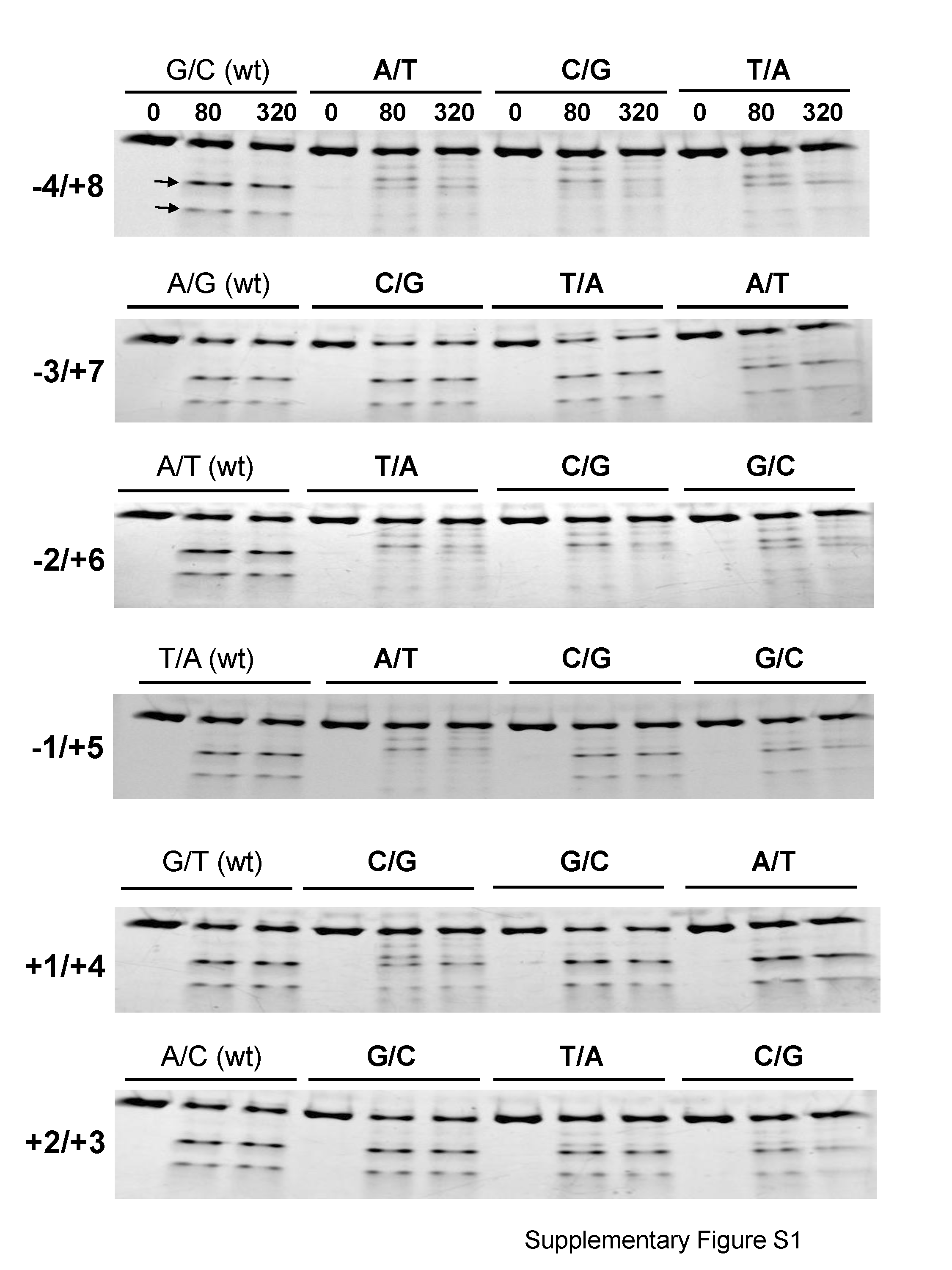

Supplement: Supplementary Data [file supp_gkt696_nar-01034-f-2013-File015.tif]

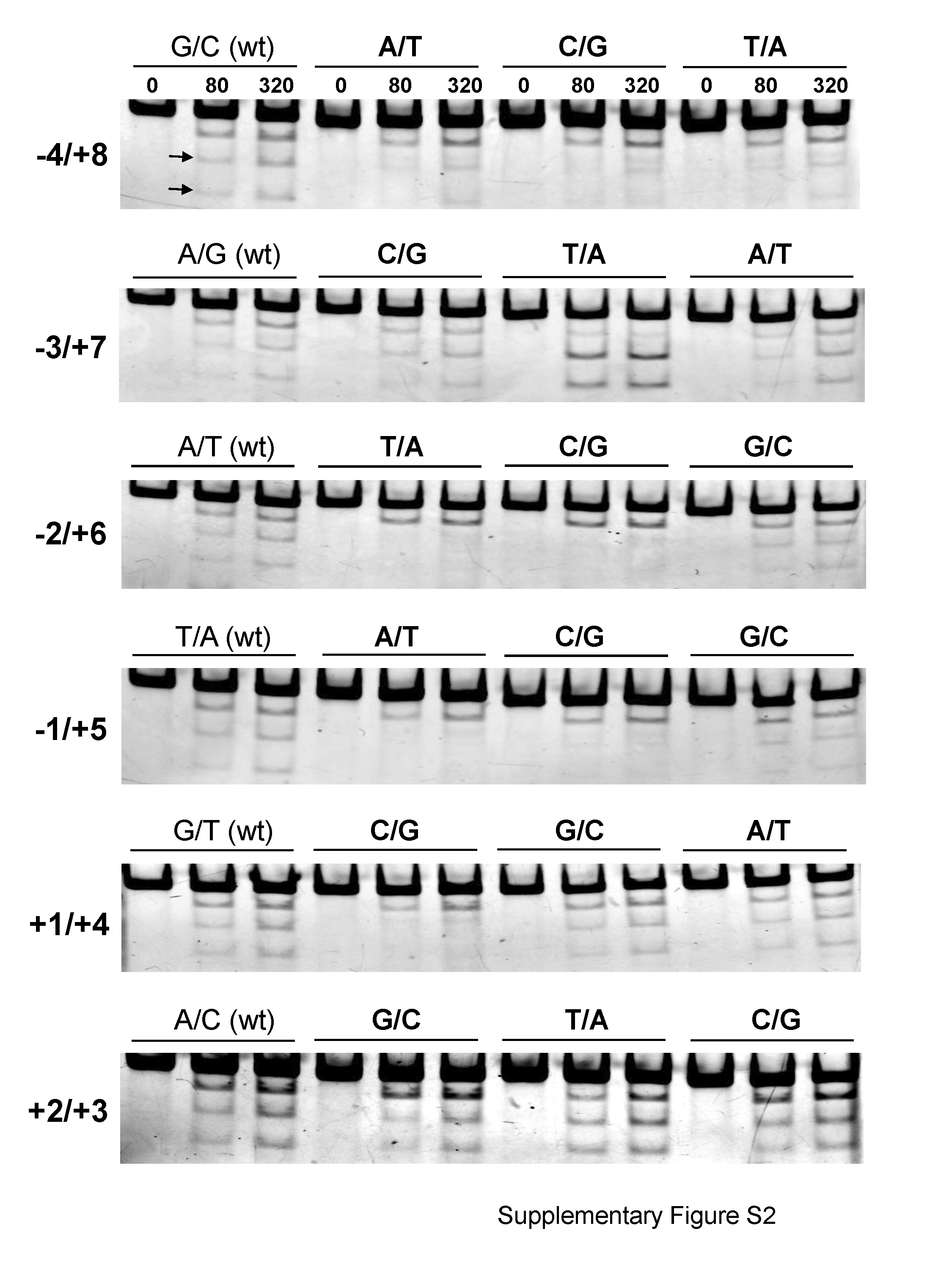

Supplement: Supplementary Data [file supp_gkt696_nar-01034-f-2013-File016.tif]

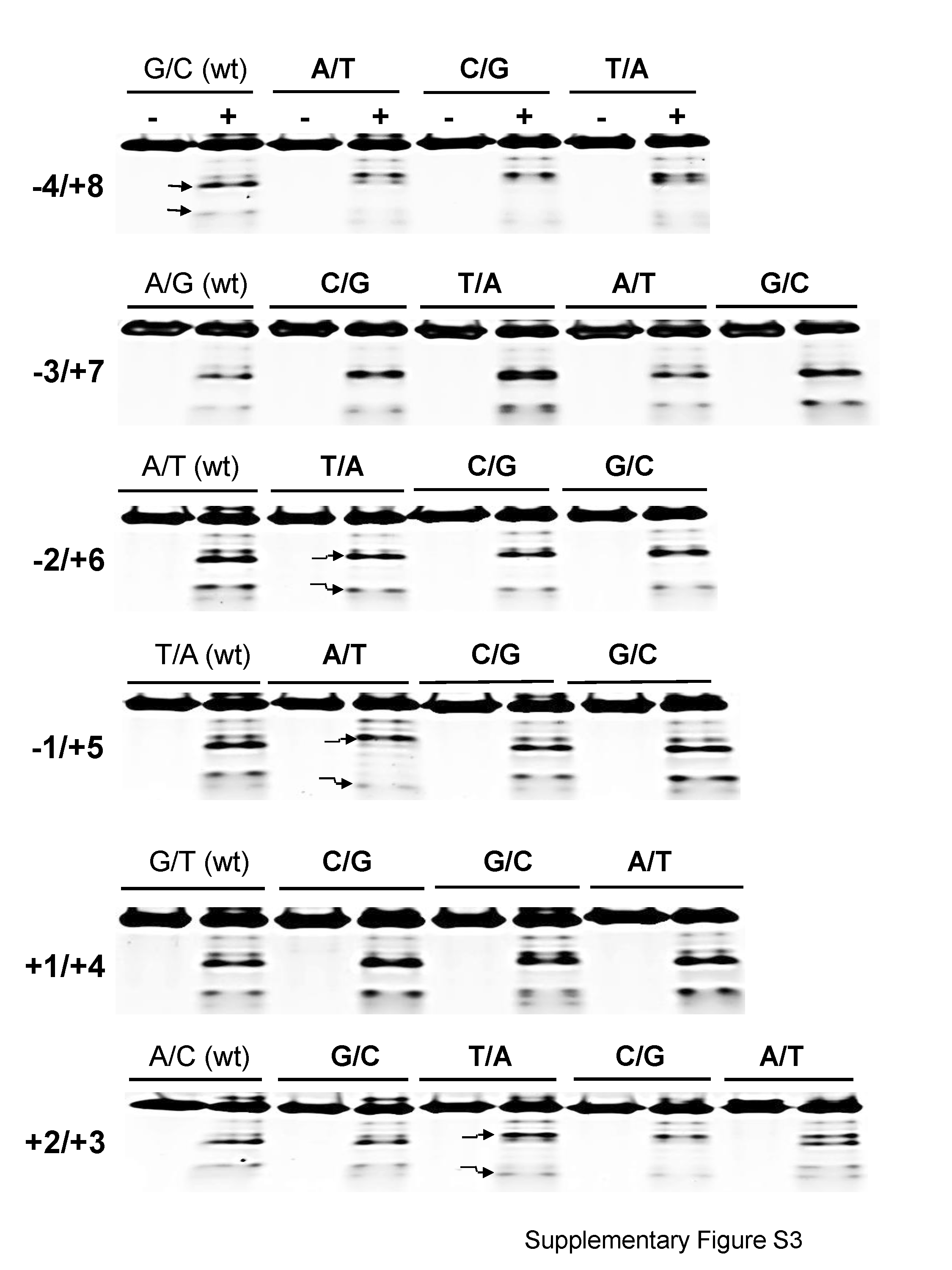

Supplement: Supplementary Data [file supp_gkt696_nar-01034-f-2013-File017.tif]

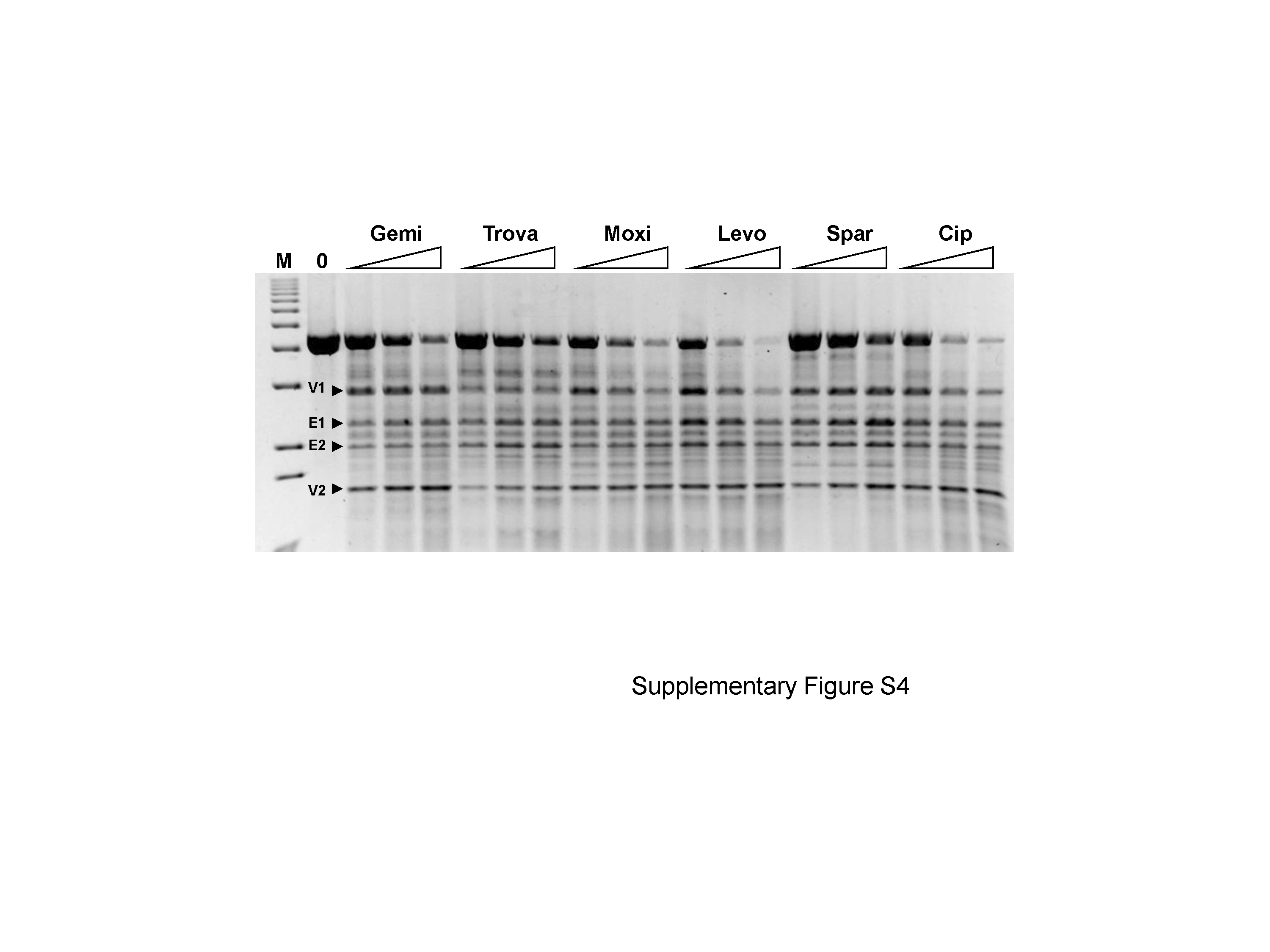

Supplement: Supplementary Data [file supp_gkt696_nar-01034-f-2013-File018.tif]

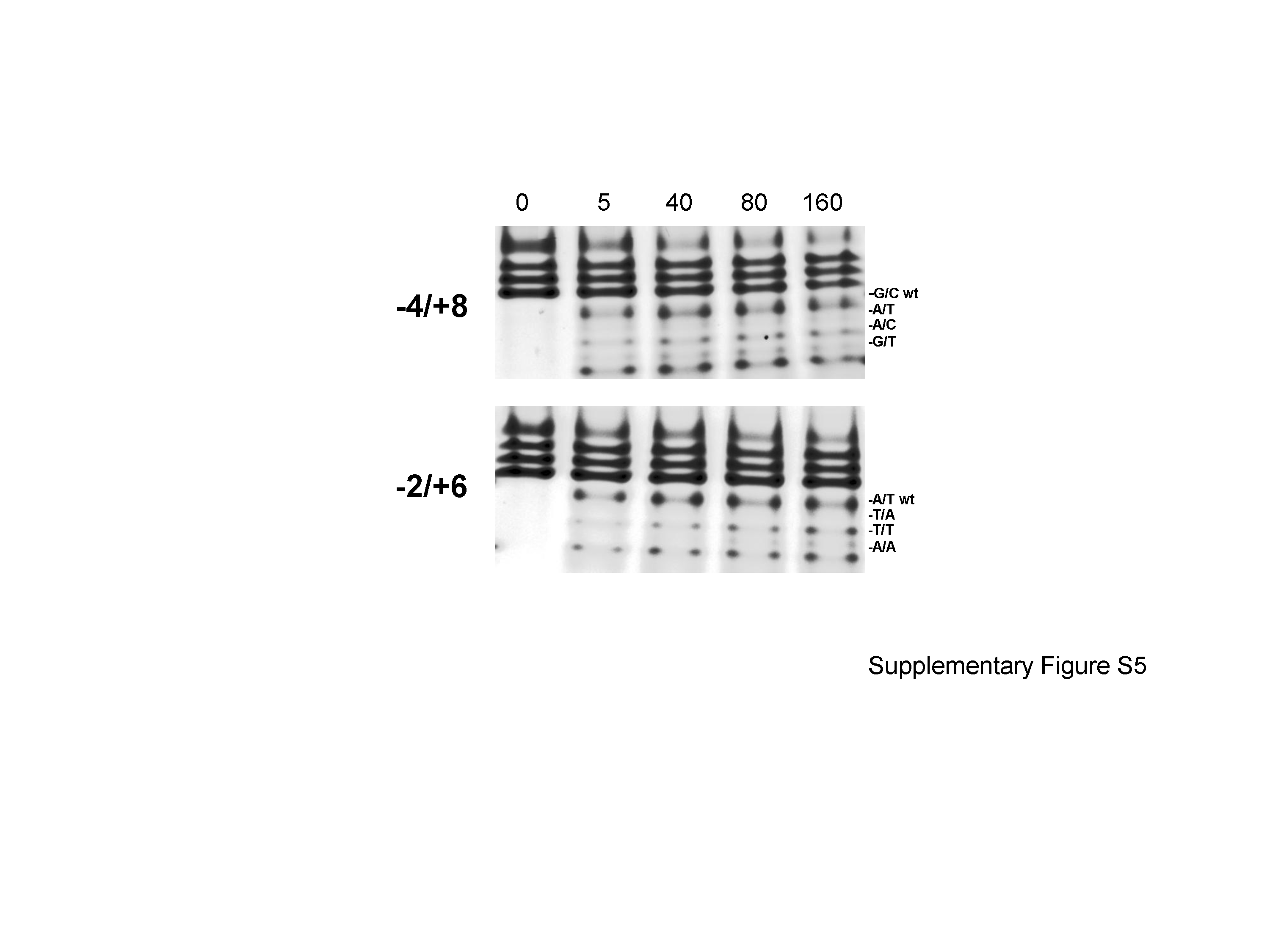

Supplement: Supplementary Data [file supp_gkt696_nar-01034-f-2013-File019.tif]
